# Open‐Source Artificial Intelligence in Small‐Bowel Capsule Endoscopy—Visual Assistance is Promising, But Not Yet a Standard of Care

**DOI:** 10.1002/deo2.70359

**Published:** 2026-06-15

**Authors:** Ervin Toth, Anastasios Koulaouzidis

**Affiliations:** ^1^ Department of Gastroenterology Skåne University Hospital Lund University Malmö Sweden; ^2^ Department of Gastroenterology Pomeranian Medical University (PUM) Szczecin Poland; ^3^ Department of Clinical Research University of Southern Denmark (SDU) Odense Denmark; ^4^ Surgical Research Unit Odense University Hospital and Svendborg Hospital Svendborg Denmark

**Keywords:** artificial intelligence, capsule endoscopy, diagnostic errors, gastrointestinal hemorrhage, small intestine

To the Editor,

We read with interest the study by Miyazono et al. [1] evaluating the open‐source SEE‐AI model for small‐bowel capsule endoscopy (SBCE). Improved lesion detection and shorter interpretation time are encouraging, especially for suspected SB bleeding. SEE‐AI is attractive because it overlays bounding boxes on the full video rather than selected frames, preserving human review while reducing visual search burden. However, caution is needed before AI‐assisted SBCE reading is considered standard of care (SoC) [2]. The fact that all AI‐assisted lesions had been flagged by SEE‐AI raises automation‐bias concerns: readers may become dependent on prompts, especially given known miss rates in CE reading [3]. Future studies should report whether readers still detect clinically relevant lesions in non‐flagged segments, across experts, non‐experts, and trainees. False positives also matter: SEE‐AI generated 365,959 boxes, only 36.6% confirmed lesions. For less experienced readers, this may increase cognitive load or overcalling. Given documented reader fatigue and inter‐/intra‐observer variability [4, 5], studies should report false alarms, confidence, overdiagnosis, and downstream consequences, not only sensitivity and reading time.

The reported time saving reflects interpretation time, not full workflow efficiency. SEE‐AI required video extraction, duplicate‐frame removal, cloud upload, GPU inference, and annotated‐video generation, reportedly taking about 1 h [1]. Routine adoption should therefore assess turnaround time, staff input, technical failures, data governance, cost, and platform integration. The reference standard was pragmatic, and training/validation used PillCam SB3 videos in a lesion‐enriched cohort. Cross‐platform, all‐comer studies are needed, including poor‐preparation, incomplete, and non‐bleeding examinations. SEE‐AI is an important step towards transparent AI assistance. Before routine adoption, prospective workflow‐complete studies should include readers of different experience levels and report model details, preprocessing, hardware, inference time, false positives, turnaround time, and clinical outcomes. For now, SEE‐AI is best viewed as a promising expert‐supervised aid instead of SoC.

## Author Contributions

**Anastasios Koulaouzidis** conceived and drafted the manuscript and critically revised the manuscript. All authors approved the final version. **Ervin Toth**: formal analysis, writing – review and editing, and writing – original draft. **Anastasios Koulaouzidis**: conceptualization, formal analysis, supervision, and writing – review and editing.

## Conflicts of Interest

Anastasios Koulaouzidis and Ervin Toth report industry and editorial relationships as detailed in the submitted ICMJE disclosure forms.

## Funding

The authors have nothing to report.

## Ethics Statement

This Letter to the Editor discusses previously published work and does not report original research involving human participants, animals, identifiable personal data, or patient‐level clinical data. Ethics committee approval, informed consent, and study registration were therefore not applicable.

## Artificial Intelligence Generated Content Statement

During manuscript preparation, the authors used ChatGPT, OpenAI, GPT‐5.5 Thinking, accessed May 2026, to assist with language condensation, reference ordering, and editorial formatting. The authors critically reviewed, edited, and verified all content, references, and conclusions, and take full responsibility for the final manuscript. No AI tool is listed as an author.

## Data Availability

Data sharing is not applicable to this article because no new data were generated or analyzed.
